# Data Acquisition System for* In Situ* Monitoring of Chemoelectrical Potential in Living Plant Fuel Cells

**DOI:** 10.1155/2016/6108056

**Published:** 2016-08-30

**Authors:** Fuei Pien Chee, Cheng Ann Chen, Jackson Hian Wui Chang, Ying Ying Choo, Jedol Dayou

**Affiliations:** ^1^Energy, Vibration and Sound Research Group (e-VIBS), Faculty of Science and Natural Resources, Universiti Malaysia Sabah, Jalan UMS, 88400 Kota Kinabalu, Sabah, Malaysia; ^2^Faculty of Resource Science and Technology, Universiti Malaysia Sarawak, 94300 Kota Samarahan, Sarawak, Malaysia; ^3^Borneo Marine Research Institute, Universiti Malaysia Sabah, Jalan UMS, 88400 Kota Kinabalu, Sabah, Malaysia; ^4^Preparatory Center for Science and Technology, Universiti Malaysia Sabah, Jalan UMS, 88400 Kota Kinabalu, Sabah, Malaysia

## Abstract

Photosynthesis process in plants generates numerous sources of bioenergy. However, only a small fraction is readily exploited for electrical energy. The impact of environmental factors is one of the significant physiological influences on the electrical potential of the plants. Hence, we developed a data acquisition (DAQ) system for instantaneous monitoring of electrical potential in plants and* Aloe vera* was used as a plant sample. The static response characterization, capability index (P/T), and Pearson's coefficient of correlation procedures were applied to assess the reliability of the obtained data. This developed system offers the capability of* in situ* monitoring and detecting gradual changes in the electrical potential of plants up to a correlational strength of greater than 0.7. Interpretation of the electrical signal mechanisms in the* Aloe vera* plant and the optimization of the electricity can be achieved through the application of this monitoring system. This system, therefore, can serve as a tool to measure and analyze the electrical signals in plants at different conditions.

## 1. Introduction

Existence of electrical signals in plant cells has been discovered and studied since 1873 [1–3]. The bioelectrochemical or electrophysiological signal is an essentially physical signal in plants as it is the most rapid method of long distance communication when compared with chemical signals such as hormones [4, 5]. Recently, many researchers have shown interest in plant-based energy generation that harvests electrical energy from the living plants; for example, a team of electrical engineers from the University of Washington devised a voltage boost converter for energy harvesting from the trees [6]. The use of living plants to harvest energy has been extensively investigated to generate a locally available, cost-effective, locally abundant, ecologically accepted, and environmentally friendly power source. In another development, Magcap [7] claims that they successfully developed a new method to harvest electricity from trees due to the difference in pH between tree tissues and soil.

Following this, researchers are taking photosynthesis processes more into consideration with the abundance of solar energy. A previous study found a method to generate bioelectricity through direct extraction of photosynthetic electrons by inserting a nanoelectrode into living algal cell. Ryu et al. [8] attempted to probe photosynthesis with their main focus on energy extraction. In addition, they developed a way to manipulate the proteins contained in the thylakoids and interrupt the pathway along which electrons flow before they are used to make sugars (high photoelectrochemical). Similarly, the possibility of harvesting electricity from a living plant using glucose oxidase (GOx) and bilirubin oxidase (BOD) modified electrodes during the photosynthesis process was shown [9]. Even though harvesting electrical output directly from living plants is still unpretentious, it is promising and certainly worth exploring further [10].

In the previous work, some fundamental procedures to harvest the weak electricity from living plants which consist of details of harvester selection, types of plants, and some potential applications were presented in [11]. The electrochemistry process is believed to be responsible for the mechanism of the energy production in the living plant cell, which is termed living-plant fuel cells or LFCs [12, 13]. The power output of LFCs is low; thus, in order to measure and sample the electric potential in the living plants, a monitoring system that collects low voltage and current continually is needed. In this paper, the development of this monitoring system for electrical potential measurement was established. The process of calibration was carried out to examine the performance of the system. Paired *t*-test was used to determine Pearson's coefficient of correlation which reveals the reliability of the test in the living plants.

## 2. Materials and Methods

### 2.1. Prototype Development of the Monitoring System

**Figure 1:**
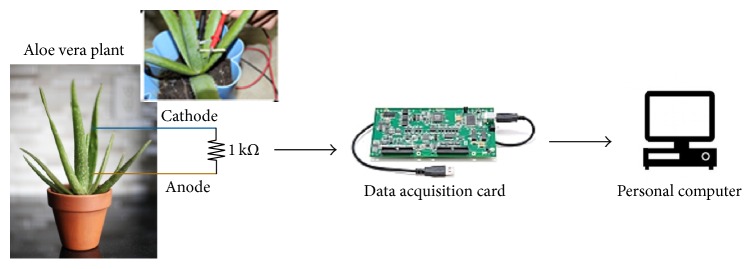
Experimental setup of living-plant fuel cell (LFC) system.

The monitoring system consists of hardware and software with data acquisition (DAQ) and processing functions. The DAQ gathers analog signals from the plant and digitalizes these data for storage, analysis, improvement, and presentation on the computer. The data acquiring method is based on the analog-to-digital converter (ADC) interface card and is controlled by the designed software developed using Visual Basic. The energy source of LFC used is the* Aloe vera* plant by embedding a pair of electrodes (copper and zinc) into it. The continuous sampling of the electric potential in the* Aloe vera* at different light intensity was taken into account. The experimental setup is as shown in Figure 1 for clarity plants where long-term measurement is performed.

### 2.2. Hardware Development

Analog-to-digital converter is used in this system and is first compared between a series of known analog signals to the analog signal to be measured until the two signals match within specific tolerance. The analog signal is then converted to digital format, which can be interpreted by the host computer. This monitoring system is designed to measure a low input voltage range (*V*_in_) with a resolution of 0.1526 mV. 16-bit A/D converter is implemented due to its higher resolution when compared to the lower bit A/D converter as shown in Table 1. This is crucial for getting an accurate and sensitive system to detect small changes in the electrical signal of the living plants. Referring to Table 1, the most significant bit (MSB) is referred to as the leftmost bit which is the bit position in a binary number having the greatest value. LSB, the least significant bit, however, is the lowest bit in a series of numbers in binary and is the one farthest to the right in a string of data. The quantization error results from the finite resolution of the A/D converter in which the amount of error is a function of the resolution of the quantizer. By definition, the quantization error of an A/D converter is half of a LSB.

**Figure 2:**
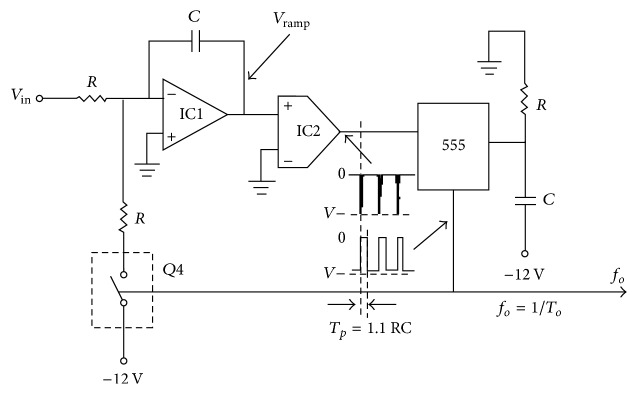
Schematic diagram of voltage-to-frequency converter (VFC) circuit.

The voltage-to-frequency converter (VFC) is used for the implementation of analog-to-digital conversion. This VFC contains two stage operational amplifiers (OA) and a precision pulse generator (NE555) as shown in Figure 2. The first stage OA is configured as a Miller Integrator together with R-C network whereas the second stage OA is operated as a comparator. The Miller effect occurs when there is an increase in the equivalent input capacitance that appeared across the OA input terminals due to the high negative gain, *A*′, of the amplifier. This increase in the input capacitance is given by

(1) $$\begin{array}{c} C=\left(\;1+A^{\prime }\;\right)\Delta C, \end{array}$$

where Δ*C* is the feedback capacitance.

The Miller effect would in turn affect the input impedance and the frequency response of the amplifier. The signal output from the first stage OA consists of excessive rounding in both of the rising edge and the falling edge of the signal during the charging and discharging process of the resistor-capacitor (R-C) network. Hence, in order to obtain a distinct level change in the signal, the signal is fed to the second stage OA which is configured as a comparator. The signal is sampled and interpreted above the reference voltage as high (“1”) and below the reference voltage as low (“0”).

**Figure 3:**
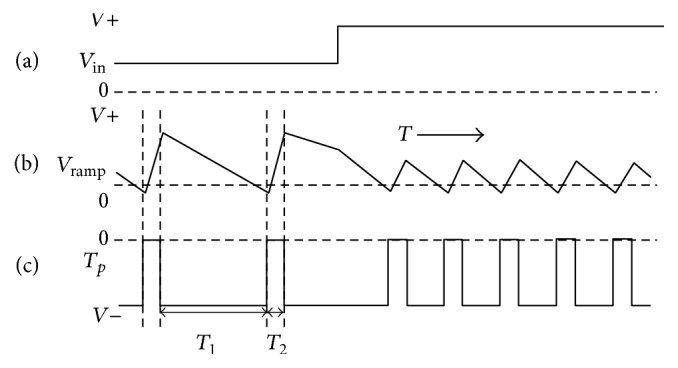
The main voltage signals: (a) output waveform of VFC, (b) integrator output, and (c) synchronous VFC output.

This process is repeated continuously, producing a digital pulse train at the VFC output as shown in Figure 3(a). The output waveform of the VFC is linearly proportional to the input voltage (*V*_in_). As the input voltage increases, the time constant (*t*) would increase which in turn causes a rise in the frequency. *V*_in_ is converted into an input current (*I*_in_); then, this current is integrated to charge the capacitor. When the signal passes the comparator threshold, a fixed charge is removed from the capacitor. The input current will continue to flow during the discharge and therefore no charge loss occurs. The waveform output of the integrator is a two-part linear ramp as shown in Figures 3(b) and 3(c). The first part of the wave lasts for time *T*_1_, which depends on the input voltage, while the second one lasts for a fixed time *T*_2_, which corresponds to the pulse width of the precision monostable. The cycle is triggered to start gain once the pulse from the monostable ends. Therefore, the output pulse rate is accurately proportional to the rate at which the integrator charges from the input. The iteration of this cycle gives a sawtooth wave at the integrator output, where the output frequency is proportional to the sum of the discharge time *T*_1_ and charge time *T*_2_.

This circuit could also be integrated with DAC as it could function as a data dispatcher to control the monitoring system by receiving commands from a personal computer. The output generated by this DAC would stay the same until it receives another value from the computer. In order to acquire and produce analog waveforms, the DAC and ADC must be activated at precise intervals. Consequently, measurement hardware had timing circuitry to produce a pulse train of a constant frequency to control the ADC and DAC.

### 2.3. Software Development

The software is developed using Visual Basic programming language which can be operated under DOS as well as under Windows operating system. This software has the ability to perform the following operations:

1. Processing the input digital signals, including arithmetical operations, comparison, ordering, and code conversion.
2. Displaying the input analog signal in numerical form.
3. Transmission of data.
4. Storage for further data handling.

The input analog signal has an infinite number of possible levels within its range. Therefore, the analog signal would then be converted into a fixed number of possible digital levels by using an encoding method. For example, to measure the changes of input voltage parameter, *V*, in the plant, the following is used:

(2) $$\begin{array}{c} V=\frac{Encoded\;\;Decimal\;\;Value}{65535}\times 10.0. \end{array}$$

65535 is a predefined constant as it is the highest number which can be represented by an unsigned 16-bit binary number as 16-bit ADC is implemented in the DAQ system.

### 2.4. Calibration and Response of the Monitoring System

Errors arise in a measurement system because no instruments are perfect and their outputs do not precisely follow their inputs. These errors can be determined through the process of calibration [14]. Calibration can be defined as comparison tests between measurements of two units: one unit is a standard device with a known value and the other unit is the device under test or is more correctly known as the device being calibrated [15, 16].

The performance of the data acquisition system used in this monitoring is first studied after development. This is to ensure the sampling of analog data recorded is accurate. Both the static response characteristics and the process capability index of this system are examined.

### 2.5. Static Response Characterization

Static calibration is performed since the output value of the DAQ system is not a function of time. MASTECH HY1803DL Variable DC Power Supply served as the standard unit in this calibration process.

The accuracy of the monitoring system is quantified through the absolute error, *e*_abs_, given by

(3) $$\begin{array}{c} e_{abs}=\left|\;true\;\;value-indicated\;\;value\;\right|, \end{array}$$

whereas the relative error, *e*_rel_, is given by

(4) $$\begin{array}{c} e_{rel}=\frac{e_{abs}}{\left|true\;\;value\right|}. \end{array}$$

Subsequently, the accuracy of the calibration, *a*_cal_, can be computed as

(5) $$\begin{array}{c} a_{cal}=1-e_{rel}. \end{array}$$

In order to achieve the accuracy of larger than 99%, several calibration tests and modifications were carried out.

### 2.6. Measurement of the Capability Index (*P*/*T*)

In any measurement, it is vital to ensure that both the measuring device and the process are able to produce output within specification limits in order to meet the technological requirements. In this paper, capability index (*P*/*T*) is used as a reference to measure the acceptable tolerance [17]:

(6) $$\begin{array}{c} \frac{P}{T}=\frac{5.15\times \sigma M}{Tolerance}, \end{array}$$

where *σM* is the standard deviation of the system and the tolerance (given as quantization error) of this 16-bit system is ±0.0763 mV as shown in Table 1.

This index is used to assess whether the system developed is statistically able to meet the specifications of six-sigma performance. A good measurement system will have a *P*/*T* ratio that is less than 10%, while merely an adequate measurement system will have a *P*/*T* ratio of less than 30% [18, 19]. The industry standard used in this calibration is 5.15 as shown in (6) which is the standard deviation accounting for 99% of the measurement system (MS) [20, 21].

### 2.7. Reliability Analysis

The stability pattern of the developed system in monitoring the weak signal of the plant is assessed using the paired *t*-test. The *t*-test is applied when there is a necessity to assess the means of two group results of two variables statistically different from each other [22]. Thus, *t*-test is carried out to determine Pearson's coefficient of correlation which reveals the reliability of the test in the living plants.

The sign of the correlation coefficient from the *t*-test determines whether the correlation is positive or negative. The magnitude of Pearson's coefficient can range from −1 to +1 whereas a value of 0 indicates that there is no association between the two variables [23]. The correlation coefficient determines the correlational strength between the tests.  Table 2 shows the crude estimates for interpreting strengths of correlations as there is no fixed rules set for this up to now [24–26].

## 3. Results and Discussion

### 3.1. Static Response Characterization

Static response characteristics were examined to ensure the sampling of analog data is accurate, which is greater than 99% [27]. The true value in the range from 0.5 V to 5.0 V was fixed from standard device, MASTECH HY1803DL Variable DC Power Supply. The calibration data of the developed system is as shown in Table 3 and the average percentage of accuracy achieved is 99.97615% with the lowest accuracy at 99.87793%. This means that the system meets the specified accuracy with an error of ±0.02385%.

### 3.2. Capability Index (*P*/*T*) of the Monitoring System

The capability index (*P*/*T*) of the system was analyzed to ensure that it is able to meet the specifications of six-sigma performance, which has a *P*/*T* ratio of less than 10%. The MASTECH HY1803DL Variable DC Power Supply was connected to a rheostat to control the desired output voltage. The known value of output voltage was then measured using the monitoring system. In this case, a small input current at a range between 1 mA and 10 mA is used as the system is priority developed for measuring weak signal in living plant.  Table 4 shows the computation of mean and *σM* of the system at *I* = 5 mA and *R* = 250 Ω.

The average of the mean and of the standard deviation of the 10 trials is 1.229731 and 0.000115, respectively. Substitution of these values to (6) yields a *P*/*T* ratio of 0.0085 (0.85%) which falls within the best case of measurement capability index.

### 3.3. Reliability Analysis

In order to evaluate the reliability of the monitoring system, a living-plant fuel cell or LFC consisting of a pair of zinc-copper (Zn-Cu) electrodes embedded in* Aloe vera* leaf was used.* Aloe vera* was selected as the sample plant as the bioenergy of this plant can be exploited as electrical energy [28]. The LFC produces a small amount of electricity through an electrochemical process as described by [11–13, 28].

Table 5 compares the mean ± standard deviation and Pearson's coefficient of the trial tests on the* Aloe vera* plants. The analysis shows that the parameters revealed good reliability as Pearson's coefficient for all tests was greater than 0.7.

## 4. Monitoring of Chemoelectrical Potential in Living-Plant Fuel Cell (LFC)

A data acquisition (DAQ) system has been developed in the previous section using analog-to-digital converter principle. The system has been confirmed to be reliable for monitoring of weak electrical signal, for example, in chemoelectrical potential of living plant.

**Figure 4:**
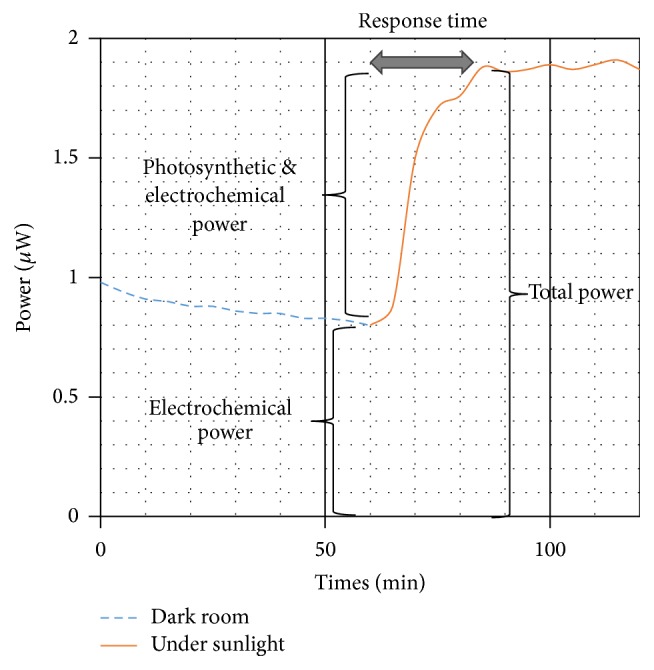
The average power profile through 1 kΩ external load of LFC in the dark room and under natural sunlight.

The system was then used to investigate the changes in the electrical signal produced by the LFC system (Zn-Cu and* Aloe vera*) under dark condition and also under sunlight illumination. Before the monitoring was initiated,* Aloe vera* was kept in a dark room for 24 hours to stabilize its metabolism. After that, the Zn-Cu electrodes were embedded into one of the leaves where the electrodes were connected in series with the DAQ system and a load resistor of 1 kΩ. The developed DAQ system was used to monitor the changes in the electrical voltage generated by the LFC. The harvested power output for the LFC system was then calculated, which was repeated three times using different leaves.  Figure 4 shows the average power output of the* Aloe vera* both in the dark and under sunlight condition for continuous monitoring of two hours. During the first hour of measurement, the average power output was recorded as about 0.9 *μ*W and gradually increased up to 1.9 *μ*W after exposure to sunlight for an hour. From Figure 4, the response time from stable electrochemistry current to stable photosynthetic current was approximately 25 minutes. There is a remarkable increment of 111% in harvested power after exposure to sunlight with an intensity of approximately 800 Wm^−2^.

## 5. Conclusion

In this paper, a data acquisition (DAQ) system was developed using analog-to-digital converter principle. The system was used to monitor weak electrical signal, for example, from plants. It was found that the electricity generated from plants increased remarkably under sunlight compared to the dark condition. This shows that the photosynthesis process increases the electricity activity in the plant and the DAQ system has successfully detected this increase. The system therefore has the potential to be used for monitoring of plant condition under environmental stress or other factors.

## Figures and Tables

**Table 1:** Characteristics of 8-bit, 12-bit, and 16-bit A/D converter.

Term	8-bit	12-bit	16-bit
Most significant bit (MSB) value	128	2048	32768
Least significant bit (LSB) value	1	1	1
Number of possible values	256	4096	65536
MSB weight	1/2	1/2	1/2
LSB weight	1/256	1/4096	1/65536
Resolution, *Q* (mv/bit), for *V*_in_ = 10 V	39.0625	2.4414	0.1526
Absolute quantization error (mV)	±19.5313	±1.2207	±0.0763

**Table 2:** Crude estimates for interpreting correlational strength.

Pearson's coefficient, *r*	Relationship
0<|*r* | < 0.3	Weak correlation
0.3<|*r* | < 0.7	Moderate correlation
|*r* | > 0.7	Strong correlation

**Table 3:** Accuracy of the monitoring system.

True value from standard unit (V)	Output data from test unit (V)				Errors		
	Trial 1 (V)	Trial 2 (V)	Trial 3 (V)	Average reading (V)	Absolute error	Relative error	Percentage of accuracy (%)
0.50	0.498169	0.501068	0.498932	0.499390	0.000610	0.001221	99.87793
1.00	1.000305	1.001068	0.998932	1.000102	0.000102	0.000102	99.98983
1.50	1.498627	1.501068	1.501373	1.500356	0.000356	0.000237	99.97626
2.00	1.999695	1.999695	2.000305	1.999898	0.000102	5.09*E* − 05	99.99491
2.50	2.500916	2.499084	2.499084	2.499695	0.000305	0.000122	99.98779
3.00	3.002594	2.999542	3.000458	3.000865	0.000865	0.000288	99.97118
3.50	3.498169	3.501831	3.498932	3.499644	0.000356	0.000102	99.98983
4.00	4.000458	3.99939	3.999542	3.999797	0.000203	5.09*E* − 05	99.99491
4.50	4.498169	4.501831	4.498932	4.499644	0.000356	7.91*E* − 05	99.99209
5.00	5.000916	4.999084	4.998016	4.999339	0.000661	0.000132	99.98678

Average							**99.97615**

**Table 4:** Mean and *σM* standard deviation of the measuring system.

Time (sec)	Voltage measurement (V)										
	Trial 1	Trial 2	Trial 3	Trial 4	Trial 5	Trial 6	Trial 7	Trial 8	Trial 9	Trial 10	Average
1	1.230009	1.227723	1.229704	1.228083	1.229856	1.230009	1.230162	1.230162	1.230162	1.230162	1.229603
2	1.230009	1.227723	1.229704	1.228083	1.229856	1.230009	1.230162	1.230162	1.230162	1.230162	1.229603
3	1.230009	1.227723	1.229704	1.228083	1.229856	1.230009	1.230162	1.230162	1.230162	1.230162	1.229603
4	1.230009	1.227723	1.229704	1.228083	1.229856	1.230009	1.230314	1.230162	1.230314	1.230314	1.229649
5	1.230314	1.227723	1.229704	1.228083	1.230182	1.230009	1.230314	1.230314	1.230314	1.230314	1.229727
6	1.230314	1.227725	1.229704	1.228214	1.230182	1.230009	1.230314	1.230314	1.230314	1.230314	1.229741
7	1.230314	1.227725	1.229704	1.228214	1.230182	1.230009	1.230467	1.230314	1.230467	1.230467	1.229786
8	1.230314	1.227725	1.229704	1.228214	1.230182	1.230009	1.230467	1.230314	1.230467	1.230467	1.229786
9	1.230467	1.227725	1.229704	1.228223	1.230467	1.230009	1.230467	1.230467	1.230467	1.230467	1.229846
10	1.230467	1.227736	1.230162	1.228223	1.230467	1.230314	1.230619	1.230467	1.230619	1.230619	1.229969

Mean	1.230223	1.227725	1.22975	1.22815	1.230109	1.23004	1.230345	1.230284	1.230345	1.230345	1.229731
*σM*	0.000183	0.0000038	0.000137	0.000067	0.000231	0.000092	0.00015	0.000114	0.00015	0.00015	0.000115

**(a):** 

Mean ± standard deviation (V)		
Trial 1	Trial 2	Trial 3
0.30095 ± 0.00196	0.30990 ± 0.00032	0.30299 ± 0.00010

**(b):** 

Paired *t*-test (∝ = 0.05)		
Pearson's coefficient		
Trial 1-trial 2	Trial 1–trial 3	Trial 2-trial 3
0.86849	0.90046	0.88853

## References

[1] Stahlberg R., Cleland R. E., Van Volkenburgh E. (2006). Slow wave potentials—a propagating electrical signal unique to higher plants. *Communication in Plants*.

[2] Fromm J., Lautner S. (2007). Electrical signals and their physiological significance in plants. *Plant, Cell & Environment*.

[3] Lautner S., Grams T. E. E., Matyssek R., Fromm J. (2005). Characteristics of electrical signals in poplar and responses in photosynthesis. *Plant Physiology*.

[4] Volkov A. G., Lang R. D., Volkova-Gugeshashvili M. I. (2007). Electrical signaling in Aloe vera induced by localized thermal stress. *Bioelectrochemistry*.

[5] Volkov A. G., Ranatunga D. R. A. (2006). Plants as environmental biosensors. *Plant Signaling & Behavior*.

[6] Hickey H. (2009). Electrical circuit runs entirely off power in trees. *UW Today*.

[7] Wacker T. Canton firm's alternative to oil: plug in to a tree.

[8] Ryu W. H., Bai S.-J., Park J. S. (2010). Direct extraction of photosynthetic electrons from single algal cells by nanoprobing system. *Nano Letters*.

[9] Flexer V., Mano N. (2010). From dynamic measurements of photosynthesis in a living plant to sunlight transformation into electricity. *Analytical Chemistry*.

[10] Hataway J., Ramasamy R. (2013). *Power Plants: UGA Researchers Explore How to Harvest Electricity Directly from Plants*.

[11] Choo Y. Y., Dayou J. (2013). A method to harvest electrical energy from living plants. *Journal of Science and Technology*.

[12] Choo Y. Y., Dayou J., Surugau N. (2014). Origin of weak electrical energy production from living-plants. *International Journal of Renewable Energy Research*.

[13] Choo Y. Y., Dayou J. (2016). Modelling of the electricity generation from living plants. *Jurnal Teknologi*.

[14] Taylor B. N. (2009). *Guidelines for Evaluating and Expressing the Uncertainty of NIST Measurement Results*.

[15] Murmann B. (2006). Digitally assisted analog circuits. *IEEE Micro*.

[16] Tseng C.-J., Chen H.-W., Shen W.-T., Cheng W.-C., Chen H.-S. (2012). A 10-b 320-MS/s stage-gain-error self-calibration pipeline ADC. *IEEE Journal of Solid-State Circuits*.

[17] Ukraintsev V. A. Effect of bias variation on total uncertainty of CD measurements.

[18] Morgan W. T. (2004). Six sigma and beyond: statistical process control. *Technometrics*.

[19] Yang Q. Reliability modeling for linear sensor systems.

[20] Sweeney S. (2007). Analysis of two-dimensional gage repeatability and reproducibility. *Quality Engineering*.

[21] Ma Y. A new method for analyzing and monitoring measurement process.

[22] Celikyilmaz A., Türksen I. B. (2009). *Modeling Uncertainty with Fuzzy Logic: With Recent Theory and Applications*.

[23] Sedgwick P. (2012). Pearson's correlation coefficient. *British Medical Journal*.

[24] Jackson S. (2012). *Research Methods and Statistics: A Critical Thinking Approach*.

[25] Weinberg S. L., Abramowitz S. K. (2008). *Statistics Using SPSS: An Integrative Approach*.

[26] Nagelkerke N. J. (1991). A note on a general definition of the coefficient of determination. *Biometrika*.

[27] Lipták B. G. (2003). *Process Control: Instrument Engineers' Handbook*.

[28] Ying C. Y., Dayou J. (2014). Increasing the energy output from living-plants fuel cells with natural photosynthesis. *Advances in Environmental Biology*.

